# A discrete dopaminergic projection from the incertohypothalamic A13 cell group to the dorsolateral periaqueductal gray in rat

**DOI:** 10.3389/fnana.2013.00041

**Published:** 2013-12-06

**Authors:** Fany Messanvi, Ellie Eggens-Meijer, Benno Roozendaal, Johannes J. van der Want

**Affiliations:** ^1^Department of Neuroscience, Section Anatomy, University Medical Center Groningen, University of GroningenGroningen, Netherlands; ^2^Department of Neuroscience, Section Medical Physiology, University Medical Center Groningen, University of GroningenGroningen, Netherlands; ^3^Department of Cognitive Neuroscience, Radboud University Medical Centre and Donders Institute for Brain, Cognition and Behaviour, Radboud University NijmegenNijmegen, Netherlands; ^4^Department of Laboratory Medicine, Children's and Women's Health, Norwegian University of Science and TechnologyTrondheim, Norway

**Keywords:** dorsolateral periaqueductal gray (dlPAG), dopamine, incertohypothalamic A11, and A13 cell groups, defensive behavior, panic

## Abstract

Several findings have indicated an involvement of dopamine in panic and defensive behaviors. The dorsolateral column of the periaqueductal gray (dlPAG) is crucially involved in the expression of panic attacks in humans and defensive behaviors, also referred to as panic-like behaviors, in animals. Although the dlPAG is known to receive a specific innervation of dopaminergic fibers and abundantly expresses dopamine receptors, the origin of this dopaminergic input is largely unknown. This study aimed at mapping the dopaminergic projections to the dlPAG in order to provide further insight into the panic-like related behavior circuitry of the dlPAG. For this purpose, the retrograde tracer cholera toxin subunit b (CTb) was injected into the dlPAG of male Wistar rats and double immunofluorescence for CTb and tyrosine hydroxylase (TH), the rate-limiting enzyme in the synthesis of dopamine, was performed. Neurons labeled for both CTb and TH were counted in different dopaminergic cell groups. The findings indicate that the dopaminergic nerve terminals present in the dlPAG originate from multiple dopamine-containing cell groups in the hypothalamus and mesencephalon. Interestingly, the A13 cell group is the main source of dopaminergic afferents to the dlPAG and contains at least 45% of the total number of CTb/TH-positive neurons. Anterograde tracing with biotinylated dextran amine (BDA) combined with double immunofluorescence for BDA and TH confirmed the projections from the A13 cell group to the dlPAG. The remainder of the dopamine-positive terminals present in the dlPAG was found to originate from the extended A10 cell group and the A11 group. The A13 cell group is known to send dopaminergic efferents to several other brain regions implicated in defensive behavior, including the central amygdala and ventromedial hypothalamus. Therefore, although direct behavioral evidence is lacking, our finding that the A13 cell group is also the main source of dopaminergic input to the dlPAG suggests that dopamine might contribute to the regulation of dlPAG-mediated defensive behaviors.

## Introduction

There is considerable evidence supporting a role for dopamine in defensive behavior and panic. Early animal studies have shown the contribution of dopamine in the general process of defensive behavior, suggesting a facilitating role for dopamine via its D2-receptors. Systemic administration of D2-receptor agonists has been shown to induce defensive and aggressive reactions whereas D2-receptor antagonists reduce these types of behavior (Maeda, 1976; Maeda et al., 1985; Puglisi-Allegra and Cabib, 1988a,b; Gendreau et al., 1998, 2000). Additionally, systemic administration of the dopamine receptor antagonists haloperidol and chlorpromazine increases the threshold of hypothalamic-induced directed attack and threat responses, while agonists such as methamphetamine and apomorphine, which enhance dopaminergic transmission, lower this threshold (Maeda, 1976; Maeda et al., 1985). Dopamine densely innervates several regions of the defensive behavior system such as the amygdala and hypothalamus (Lindvall et al., 1984; Asan, 1998). It is likely that dopamine release into these brain structures directly participates in the facilitation of defensive behavior and panic-like reactions. For instance, an activation of D2-receptors within the medial preoptic-anterior hypothalamus has been shown to induce defense-like reactions in cat (Sweidan et al., 1991).

The dorsolateral column of the periaqueductal gray (dlPAG) is another key structure of the defensive behavior system (Bandler and Shipley, 1994; Del-Ben and Graeff, 2009; Fogaça et al., 2012). It responds to the presence of proximal threat (Reiman et al., 1989; Mobbs et al., 2007) and its stimulation elicits panic attack and defensive behaviors in both human and animal subjects (Nashold et al., 1969; Bandler et al., 1985; Deakin and Graeff, 1991; Jenck et al., 1995; Adams, 2006). An interesting study from Jenck et al. (1990) demonstrated that nomifensine, a dopamine- and noradrenaline-reuptake inhibitor, enhances the aversion induced by electrical stimulation of the dorsal PAG, suggesting a role for dopamine within this region. The presence of dopamine fibers and dopamine D2-receptor in the dlPAG has been shown in rat and in cat (Mansour et al., 1990; Kitahama et al., 2000, 2007). Taken together, these findings suggest that dopamine might participate in regulating the expression of dlPAG-mediated panic-like behaviors.

A first step to answer this question is to establish a map of dopaminergic afferents to this region. Therefore, this study aimed first at investigating the existence of a dopaminergic innervation into the dlPAG by applying immunohistochemistry for tyrosine hydroxylase (TH) and dopamine-β-hydroxylase (DBH), the rate-limiting enzymes in the synthesis of dopamine and noradrenaline respectively, to distinguish between dopaminergic fibers (TH positive) and (nor)adrenergic fibers (TH and DBH positive). Subsequently, to determine the source of dopaminergic neurons projecting to the dlPAG, the retrograde tracer cholera toxin subunit b (CTb) was injected into the dlPAG, and immunofluorescence for CTb and TH was examined in several dopaminergic cell groups. The anterograde tracer biotinylated dextran amine (BDA) was used to confirm the findings obtained with the retrograde tracing experiments. We investigated the distribution and localization of dopaminergic cells projecting to the dlPAG, and showed that they originate from distinct dopaminergic cell groups.

## Experimental procedures

### Animals

Twenty male Wistar rats (250–300 g at the time of surgery) from Harlan, The Netherlands, were group-housed (2 or 3 rats per cage) and maintained on a 12-h/12-h light/dark cycle (lights on 0700-1900 h) with *ad libitum* access to food and water. All experimental procedures were in compliance with the European Communities Council Directive of 24 November 1986 (86/609/EEC) and approved by the Institutional Animal Care and Use Committee of the University of Groningen, The Netherlands.

### General surgical procedures

Nineteen rats were anesthetized by isoflurane inhalation (5% in 800 ml/min oxygen) and positioned in a stereotaxic apparatus (Kopf Instruments, Tujunga, CA, USA), fitted with a nose cone allowing for the continuous administration of anesthetic. After the rat was placed in the stereotaxic apparatus, the percentage of isoflurane was lowered to 2.5%. After cleaning of the surface, an incision was made in the skin covering the skull, and burr holes were drilled in the skull overlying the region of interest. Tracer injections were made stereotaxically, using coordinates taken from the rat brain atlas of Paxinos and Watson (1998). Subsequently, the wound was sutured and cleaned. After surgery, the rats received a subcutaneous injection of the non-steroidal analgesic carprofen (4 mg/kg; Pfizer, NY, USA) and were allowed to recover from anesthesia in individual cages placed on a heating pad for 1 h. The following day, they received a second injection of carprofen (4 mg/kg).

#### Injection of retrograde tracer

CTb (2%, List Biological Laboratories, Campbell, CA, USA), dissolved in distilled water, was targeted into the right dlPAG (coordinates: 6.3–6.6 mm posterior to Bregma, 1.6 mm lateral to the midline, 4.4–4.9 mm from the surface of the dura mater, angle 15°) of 13 animals. The tracer was delivered into the targeted area through a glass micropipette, either by pressure injection (pipette tip diameter 20–30 μm; volume ranged between 20 and 75 nl) using an automated air-pressure system (World Precision Instruments PV830, Sarasota, FL, USA), or by iontophoresis (pipette tip diameter 10–20 μm) using a 5–μA positive-pulsed current (7 s on/off circles for 20 min). To avoid leakage of tracer along the pipette track, the pipette was left in place after the pressure injection for 5–10 min, and after the iontophoresis injection for 10 min with negative current. Out of 13 cases, six cases (R22, R23, R25, R26, R29, R30) were selected for the quantitative analysis.

#### Injection of anterograde tracer

In order to certify the projections found with the above-described retrograde tracing technique, anterograde projections originating from the region that contained most of the double-labeled cell bodies for CTb and TH were analyzed. The anterograde tracer BDA (10,000 mW, 10%; Molecular Probes, Carlsbad, CA, USA) was stereotaxically injected into the A13 dopaminergic cell group located in the medial zona incerta (coordinates: 2.4 mm posterior to Bregma; 2.1 mm lateral to the midline; 7.9 mm from the surface of the dura mater; angle 10°) by pressure injection following the same procedure as described in the section above (pipette tip diameter 20–30 μm; volume ranged between 20 and 75 nl). Out of six cases, two cases (F424 and F425) with injections that covered the entire A13 cell group were selected for further qualitative analysis.

#### Perfusion and tissue collection

Following a postsurgical survival period of 1 week, rats were anesthetized with an overdose of sodium pentobarbital (60 mg/kg, i.p.) and perfused transcardially with 50 ml of heparinized (5000 IE/ml) 0.9% (w/v) saline (Leo Pharma, Breda, The Netherlands), followed by 300 ml of 4% (w/v) paraformaldehyde in 0.1 M phosphate buffer (PB, pH 7.4). The brains were removed, postfixed at 4°C for 24 h, and then transferred to 25% sucrose in 0.1 M PB, pH 7.4 at 4°C for 48 h. An incision mark was made on the right side of the brain, into which tracers were delivered, to allow for ipsi- and contralateral distinction of projection patterns. Thirty-μm-thick frozen coronal sections of the entire brain were cut on a cryostat and collected in five series in Tris-buffered saline (TBS) with sodium azide (0,01%) and stored at 4°C until use.

To determine the distribution of TH and DBH in the rat PAG, one rat was anesthetized and sacrificed according to the same procedure. The brain tissue was also collected, sectioned and stored following the same procedure.

### Immunohistochemistry

#### TH and DBH immunohistochemistry

Brain sections containing the dlPAG, obtained from the rat that did not receive tracer injections, were pretreated with 1% sodium borohydrate for 15 min, rinsed in TBS, treated with 1% hydrogen peroxide in TBS for 1 h, rinsed again in TBS, preincubated in 5% normal donkey serum (Vector Laboratories, Burlingame, CA, USA) in 0.3% Triton X-100/TBS for 30 min, and then incubated with a polyclonal rabbit antiserum for TH (1:1,000; Millipore, Bedford, MA, USA; **catalog nr: AB152**) or a monoclonal mouse antiserum for DBH (1:400; Millipore, Bedford, MA, USA; **catalog nr: MAB308**) in 1% normal donkey serum in 0.3% Triton-X/TBS overnight at 4°C. The next day, the sections were rinsed in TBS for 90 min, incubated with biotinylated donkey anti-rabbit or donkey anti-mouse (1:1,000; Jackson ImmunoResearch, West Grove, PA, USA) and rinsed in TBS for 90 min. Subsequently, the sections were incubated with avidin-biotin peroxidase complex from the Vectastain Elite Kit (Vector Laboratories, Burlingame, CA, USA). Tissue was rinsed in TBS for another 90 min and the peroxidase product was visualized by transferring sections into 0.022% diaminobenzidine (Sigma-Aldrich, USA) and 0.003% hydrogen peroxide in TBS for 5 min. The reaction step was terminated by thorough rinsing in TBS. The sections were then mounted on glass slides, air-dried, dehydrated in a gradient of alcohol, cleared in xylene, and cover slipped with DePeX mounting medium (Gurr BDG, Poole, UK).

#### Visualization of injection sites

In order to visualize the injection sites, dlPAG sections obtained from tracer-injected rats were processed following the procedure mentioned above. For this staining, we used a polyclonal goat antiserum for CTb (1:10,000; List Biological Laboratories, Campbell, CA, USA; **catalog nr: 703**) as primary antibody, biotinylated rabbit anti-goat (1:1,000; Jackson ImmunoResearch, West Grove, PA, USA) as secondary antibody, and normal rabbit serum instead of normal donkey serum.

### Immunofluorescence

#### CTb and TH immunofluorescence

In order to identify the location of retrogradely labeled neurons in the various dopaminergic regions, double labeling for CTb and TH was performed. The dopaminergic cell groups have been well described and classified, and have locations very distinct from the (nor)adrenergic cells groups (Dahlstrom and Fuxe, 1964; Hökfelt et al., 1984). Therefore, we chose TH as marker to visualize putative dopaminergic neurons. Three out of five series of sections of each brain having received CTb injections were preincubated with 5% normal donkey serum in 0.3% Triton X-100/TBS for 30 min, and then incubated with a cocktail of polyclonal rabbit antiserum for TH (1:1,000; Millipore, Bedford, MA, USA) and polyclonal goat antiserum for CTb (1:10,000) diluted in 1% normal donkey serum in 0.3% Triton X-100/TBS overnight at 4°C. The next day, the sections were rinsed in TBS for 90 min, and incubated with Alexa 568 conjugated donkey anti-goat and Alexa 488 conjugated donkey anti-rabbit (1:500; Jackson ImmunoResearch Laboratories, West Groves, PA, USA) diluted in 1% normal donkey serum in 0.3% Triton X-100/TBS for 1 h. After the last incubation step, sections were rinsed again in TBS for 90 min, mounted on gelatin-coated glass slides, air-dried, cover slipped with Vectashield mounting medium (Vector Laboratories, Germany) and stored in the dark.

To control for non-specific staining of the secondary antibodies, the primary antibodies were omitted or replaced with non-immune serum in some control sections. Virtually no staining was observed in those sections.

#### BDA and TH immunofluorescence

In order to certify the findings of the retrograde tracing study and visualize the dopaminergic fibers in the dlPAG that originate from the A13 cell group, double labeling for BDA and TH was performed. Sections containing the area of the A13 and the dlPAG were examined to localize double-labeled soma at the injection sites (A13 dopaminergic cell group) and double-labeled fibers in the target area (dlPAG). A one-in-five series of sections was processed following the same procedure as described in the previous paragraph. The same TH (1:1,000) antibody was used, together with the same secondary antibody or Alexa 633 conjugated donkey anti-rabbit. BDA labeling was detected with TRITC or Alexa 488 conjugated streptavidin (1:500; Jackson ImmunoResearch Laboratories, West Grove, PA, USA).

### Analysis

The CTb injection sites of the selected cases (R22.R23.R25.R26. R29.R30) were drawn using bright field and dark field illumination with a Zeiss Stemi SV11 microscope. Injection sites and (double)-labeled cells were visualized with a Leica DM 4000B fluorescence microscope and double-labeled fibers were examined with a Leica TCS SP2 confocal microscope. For the CTb/TH double-staining experiments, CTb-retrogradely labeled and TH-positive neurons were viewed with excitation lights of 560 nm wavelength (Leica TX2 filter block) and 480 nm wavelength (Leica L5 filter block), respectively. For BDA/TH double-stained sections, BDA labeling was visualized with a TX2 filter and TH with an L5 filter. Neurons and fibers showing the same morphology, position and orientation under the two different filters for the detection of CTb and TH, or BDA and TH, in the same focal plane, were considered to be double labeled. Photographs were taken with a Leica digital camera DFC 420C. The two-channel readings for green and red fluorescence were merged by using Adobe Photoshop, allowing for adjustments in contrast and brightness.

### Double-labeled cell counting

Double-labeled neurons were counted in three-in-five series of sections for each brain in the different dopaminergic cell groups. In order to estimate the total number of double-labeled neurons in each brain, the number for each group and for each subject was first multiplied by 5/3 (for the dlPAG cases) or 5/1 (for the control case) to correct for the uncounted sections. The results were then multiplied by the Abercrombie correction factor in order to correct for split nuclei and potential double counting (Abercrombie, 1946). The formula is:

A=[t/(t+x)]

Where *t* equals the thickness of the section (30 μm), and x equals the average diameter of the soma of the dopaminergic neuron. To measure the diameter of TH-immunoreactive neurons, double-labeled neurons were captured at high magnification, and the images were imported into Image J (Bethesda, MD, USA). Using this software, we determined the size of TH-containing soma and calculated an average diameter for each dopaminergic group of each animal. The value of the correction factors ranged from 0.63 to 0.72.

The CTb/TH-positive cells were plotted on sections taken from the atlas of Paxinos and Watson (1998). The quantification was expressed in percentage of the total number of labeled cells.

## Results

### TH and DBH immunoreactivity in the PAG

**Figure 1:**
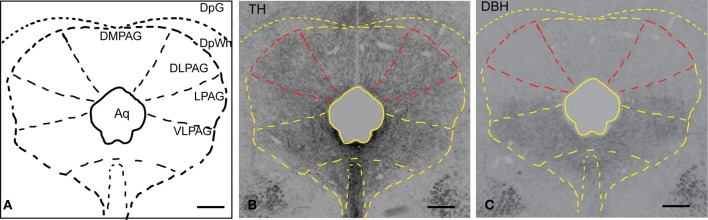
**Distribution of TH and DBH immunoreactivity in the PAG. (A)** Schematic representation of the PAG and adjacent nuclei. **(B)** Micrograph of a coronal section showing TH-labeled fibers and terminals in the PAG and deep layers of the SC. The dlPAG is outlined in red; the subdivisions of the PAG are depicted in yellow. **(C)** Micrograph of a coronal section showing DBH-labeled fibers and terminals in the PAG and deep layers of the SC. The dlPAG is outlined in red. Comparison between Figures 1B,C suggests that dopamine is more abundant than noradrenaline in the dlPAG. Scale bar is 200 μm. For abbreviations, see list.

In order to examine the presence of putative dopaminergic fibers in the dlPAG, sections were stained with an antibody against TH and DBH (the rate-limiting enzyme in the synthesis of noradrenaline). Figure 1A shows a schematic representation of the PAG observed in the stained sections. Figure 1B shows the distribution of TH-immunoreactive fibers in the whole PAG. We observed a uniform and relatively dense presence of TH immunoreactivity in the different columns of the PAG. As shown in Figure 1C, DBH-immunoreactive fibers are predominant in the ventral part of the PAG, whereas they are scarce in the dorsal part of the PAG. A comparison between TH and DBH immunoreactivity in the dlPAG (outlined in red in Figures 1B,C) indicates that TH-containing fibers are much more abundant than DBH-containing fibers, indicating that dopamine is more abundant than noradrenaline in the dlPAG.

### Origin of TH immunoreactive fibers in the dlPAG

To determine the origin of the dopaminergic innervation of the dlPAG, the retrograde tracer CTb was injected into the dlPAG, and double immunofluorescence for TH and CTb was applied. Out of the 13 rats that underwent stereotaxic surgery, six rats were selected for analysis.

#### CTb injection sites in the dlPAG

**Figure 2:**
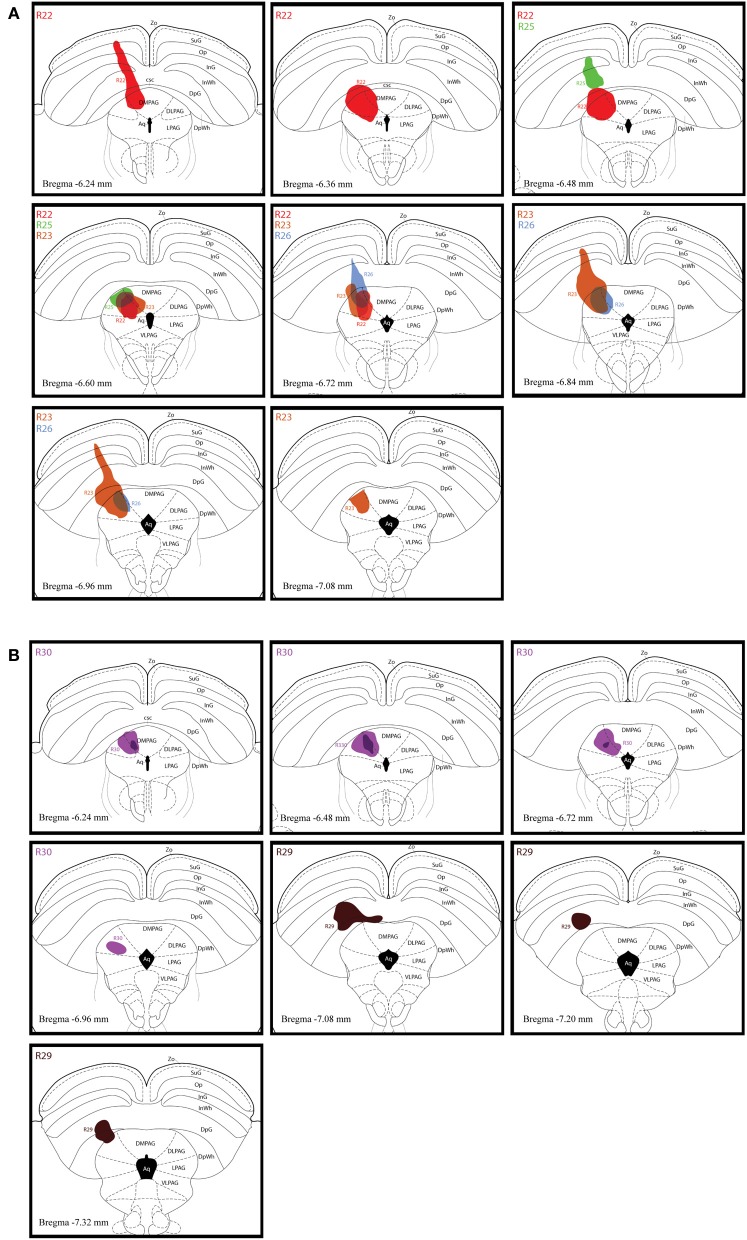
**Injection sites of retrograde tracer experiments. (A)** Schematic drawings representing CTb deposits by pressure injections along the rostro-caudal extent of the dlPAG and the SC. The four cases are represented by different colors (R22 in red, R23 in brown, R25 in green and R26 in blue). **(B)** Schematic representation of CTb iontophoresis deposits in the dlPAG. R30 is in purple and R29 in dark brown. The injection made in case R29 was placed in the deep layers of the SC and did not include the dlPAG. This case was used as a control injection. For abbreviations, see list.

Figure 2A shows schematic drawings of pressure CTb injection sites in the dlPAG, Figure 2B shows an iontophoretic CTb injection. In five cases the injection was targeted to the dlPAG. In three cases (R22, R23 and R30), the tracer deposits involved a large proportion of the rostro-caudal extent of the dlPAG. In case R22, the CTb deposit was mainly located in the rostral and intermediate dlPAG. The tracer was spread into the superior colliculus (SC) at the level of the rostral dlPAG, and also into the dorsomedial PAG (dmPAG). In case R23, the injection site covered the intermediate and caudal parts of the dlPAG as well as the SC at the level of the intermediate/caudal part of the dlPAG. In case R30, the CTb deposit was located within the dlPAG with only minor spread into the dmPAG. In two other cases (R25 and R26), the injection sites were more restricted. For case R25, the tracer deposit was contained within the rostral-intermediate dlPAG and SC. In case R26, the injection site was located in the intermediate-caudal dlPAG and the deep layers of the SC.

In case R29, the deposit was essentially located within the SC. This case was used as a control.

#### Distribution and quantification of CTb/TH double-labeled neurons in the dopaminergic cell groups

CTb/TH double-labeled neurons were found in several dopaminergic cell groups. In Table 1, the calculated numbers of double-labeled neurons in the different dopaminergic cell groups are reported for all the cases. In addition, the relative contribution of each dopaminergic cell group is indicated as a percentage of the total number of CTb/TH neurons.

**Table 1:** **Calculated numbers of putative dopaminergic neurons that project to the dlPAG and relative contribution of the different cell groups**.

**Dopaminergic cell groups**	**cases**	**PAGdl injections**					**Control injection**
		**R22**	**R23**	**R25**	**R26**	**R30**	**R29**
A15		0 (0%)	2 (2%)	0 (0%)	2 (1%)	0 (0%)	0 (0%)
Ventral hypothalamus							
A14		18 (10%)	3 (3%)	1 (3%)	20 (15%)	0 (0%)	0 (0%)
Periventricular hypothalamus							
A13		110 (59%)	51 (50%)	20 (69%)	61 (45%)	28 (78%)	3 (50%)
Medial zona incerta							
A11		16 (9%)	12 (12%)	8 (28%)	14 (10%)	8 (22%)	0 (0%)
Dorso-posterior hypothalamus Periventricular gray							
A10		40 (22%)	31 (30%)	0 (0%)	36 (26%)	0 (0%)	3 (50%)
Posterior hypothalamic area Rostral linear raphe nucleus Ventral periaqueductal gray							
A9		0 (0%)	3 (3%)	0 (0%)	4 (3%)	0 (0%)	0 (0%)
Substantia nigra							
TOTAL		182	104	29	137	36	6

The measurements were obtained from 3:5 series for the cases with dlPAG injections, and from 1:5 for the control case (R29). The estimated numbers were calculated by using the Abercrombie correction. For each case, the relative contribution of the different dopaminergic cell groups was calculated as a percentage of double-labeled neurons estimated in the specific region, divided by the estimated total number of double-labeled neurons.

The distribution pattern and proportion of double-labeled neurons were relatively constant among the different cases, although there were noticeable differences that might be explained by a variation in injection size and/or spread of tracer along the rostro-caudal extent of the dlPAG.

**Figure 3:**
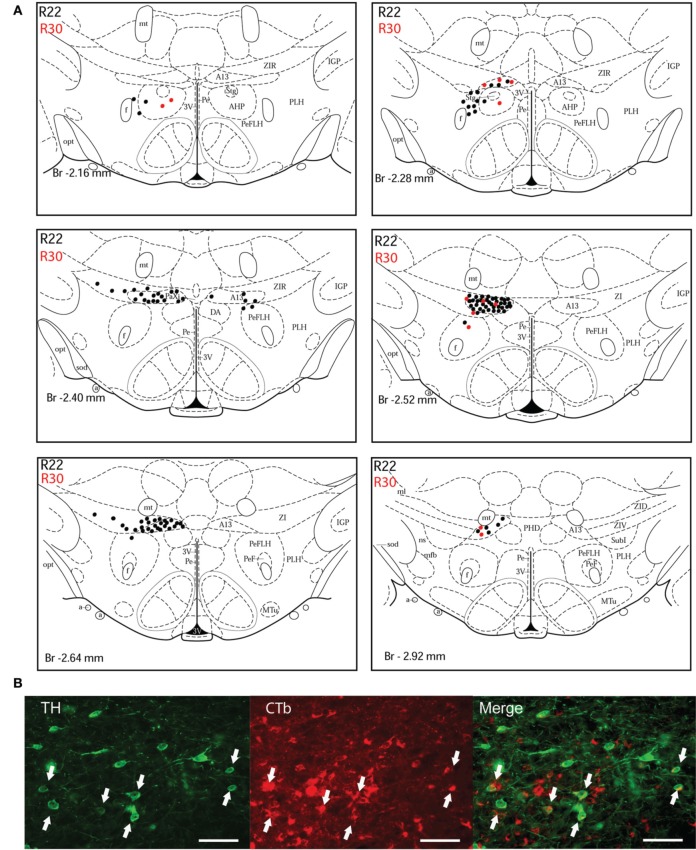
**Double-labeled neurons in the A13 dopaminergic cell group projecting to the dlPAG. (A)** Diagrammatic representation of the distribution of CTb/TH-positive cells in the A13 cell group, cases R22 (black dots) and R30 (red dots). Figures modified from Paxinos and Watson (1998). **(B)** Fluorescence micrographs showing TH-labeled (green) and CTb-labeled neurons (red) in the A13 group in case R22. Double-labeled neurons appear yellow in the merged image (white arrows). Scale bar is 60 μm.

**Figure 4:**
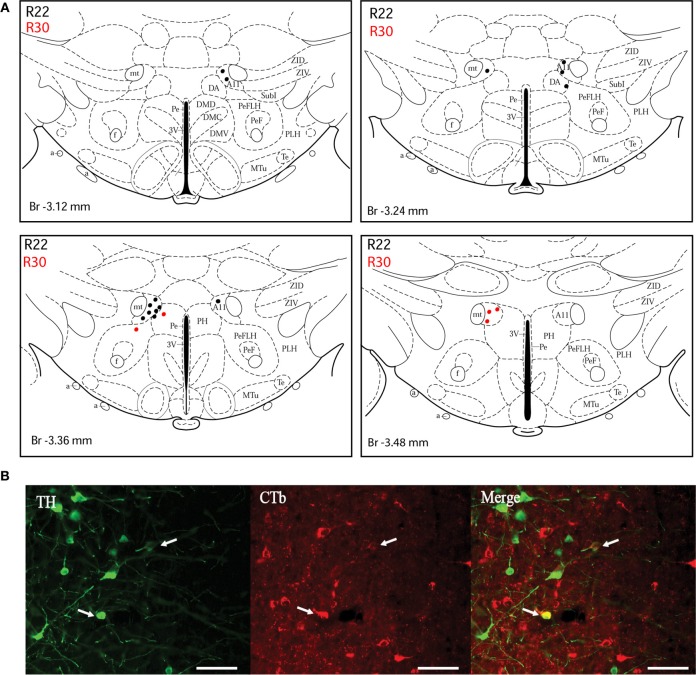
**Double-labeled neurons in the A11 dopaminergic cell group projecting to the dlPAG. (A)** Distribution of CTb/TH-positive cells in the A11 cell group, cases R22 (black dots) and R30 (red dots). Figures modified from Paxinos and Watson (1998). **(B)** Fluorescence micrographs showing TH-labeled (green) and CTb-labeled neurons (red) in the A11 group in case R22. Double-labeled neurons appear yellow in the merged image (white arrows). Scale bar is 50 μm.

**Figure 5:**
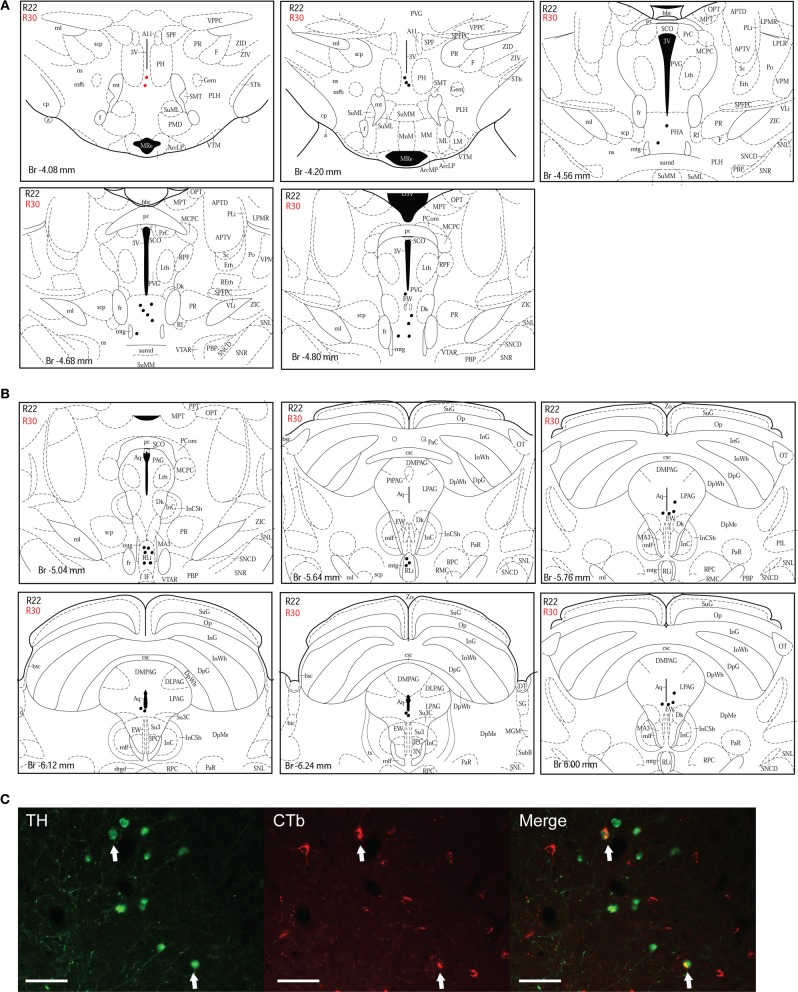
**Double-labeled neurons in the A10 dopaminergic cell group projecting to the dlPAG. (A)** Distribution of CTb/TH-positive cells in the posterior hypothalamic area (PHA), case R22 (black dots) and R30 (red dots). Figures modified from Paxinos and Watson (1998). **(B)** Distribution of double-labeled neurons in the rostral linear nucleus of the raphe and the ventral PAG. Figures modified from Paxinos and Watson (1998). **(C)** Fluorescence micrographs showing TH-labeled (green) and CTb-labeled neurons (red) in the rostral linear nucleus in case R22. Double-labeled neurons appear yellow in the merged image (white arrows). Scale bar is 100 μm.

In order to demonstrate the distribution of the putative dopaminergic neurons projecting to the dlPAG, two cases (R22 and R30) were chosen for illustration (Figures 3–5). Due to their restricted injection sites, they provide a relatively complete covering concerning the origin of dopaminergic afferents of the dlPAG.

***A13 cell group***. The incertohypothalamic dopaminergic neuron group (A13) is located in the medial part of the zona incerta (mZI), in the vicinity of the A14 cell group. In all cases, the A13 cell group was the dopaminergic cell group that contained the highest number of CTb/TH-positive cells, representing 60% of the total number of double-labeled cells in R22, 50% in R23, 59% in R25, 45% in R26, and 78% in R30. CTb/TH-positive neurons were abundant in the intermediate A13 cell group, whereas only few double-labeled neurons were found in the rostral and caudal parts. Most of these cells were located at the ipsilateral side of the tracer injection. Some contralateral double-labeled neurons were found (Figure 3A). This was observed also in cases R23 and R26 which contain higher amounts of tracer (not shown). Fluorescent images of the A13 cell group show dense clusters of labeled somata, especially for TH immunostaining with single CTb and double-labeled cells for CTb and TH (Figure 3B).

***A11 cell group***. In case R22, there were equal numbers of double-labeled cells found ipsilaterally and contralaterally to the injection site (Figure 4A). In case R30, very few neurons (we estimated a total of 8 neurons) were double labeled and all of them were found ipsilateral to the injection site (Figure 4A). In contrast, in case R25 (in which the injection site was more rostral) and case R30 (in which the CTb deposit was light), the A11 cell group contained the second highest number of CTb/TH-positive cells (28% of the total number of double-labeled neurons for R25, and 22% for R30) with no double-labeled neurons found in the extended A10 cell group. The A11 dopaminergic cell group appears at Bregma level −3 mm where it intermingles with the caudal A13 group. However, dopaminergic cells within the A11 cell group (Figure 4B) can easily be distinguished by their large somata. A few CTb/TH-positive neurons were located in this group.

***A10 cell group***. Double-labeled neurons were found in three distinct parts of the extended A10 cell group: The posterior hypothalamic area (PHA) illustrated in Figure 5A; and the rostral linear nucleus of the raphe (RLi) and the ventral periaqueductal gray (vPAG) represented in Figure 5B. Labeled neurons were equally distributed among those three sites. Rostrally, they were located ventral to the third ventricle between the mammillotegmental tract and the fasciculus retroflexus and extended into the RLi and the vPAG. The ventral tegmental area was devoid of any retrogradely labeled neurons. Figure 5C shows double-labeled neurons in the RLi of case R22. In three cases (R22, R23, and R26), the A10 cell group was the second source of CTb/TH-positive cells with respectively 22, 30, and 26% of the total number of double-labeled neurons. These were the cases in which the injection site was quite large and/or involved the intermediate part of the dlPAG.

***Other cell groups***. In all cases, except R30, double-labeled neurons were also observed in the A14 group. Very few double-labeled cells were found in the A15 and A9 (substantia nigra) groups in R23 and R26. Their injection sites shared similarities with tracer spread into the intermediate/caudal dlPAG and the SC.

***Control case***. In case R29, the tracer deposit was located in the deep layers of the SC at the level of the intermediate dlPAG. By using the same calculations as for dlPAG cases, we found an estimated number of 6 double-labeled neurons. Double-labeled cells were found in equal numbers in the A13 and the A10 cell groups (data not shown).

### Dopaminergic projection from A13 to the dlPAG

**Figure 6:**
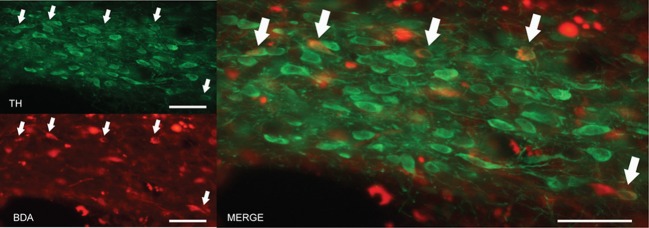
**BDA injection site in the area of the mZI, including the A13 cell group.** Images from fluorescent microscopy show neurons containing the anterograde tracer (in red) and TH in (green). Double-labeled cells are indicated by arrows and appear in yellow. Scale bar is 30 μm.

**Figure 7:**
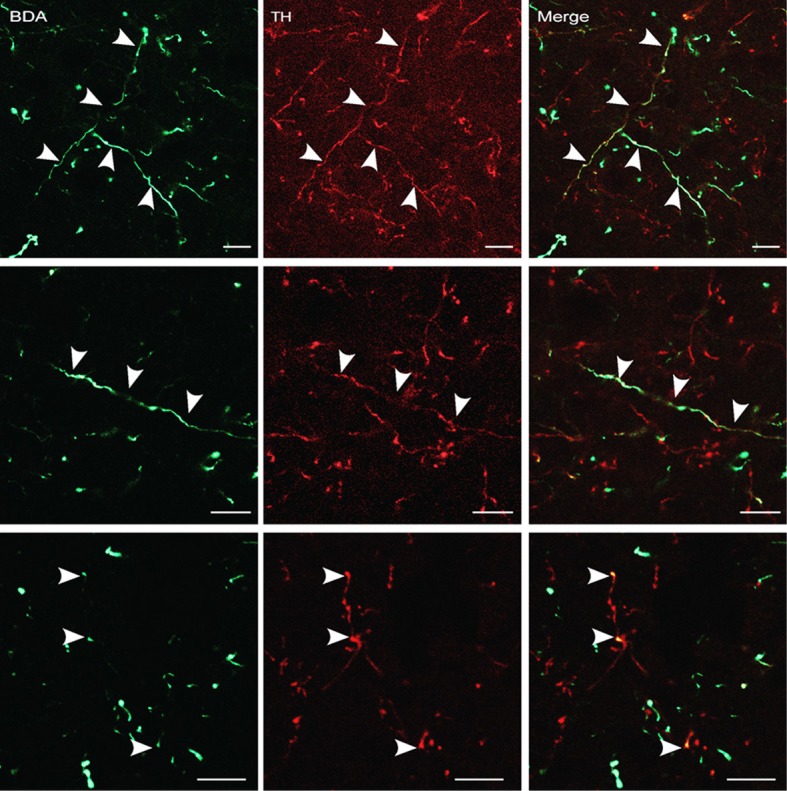
**BDA-labeled terminals and terminal boutons in the dlPAG originating from the A13 dopaminergic cell group.** Upper panel: images from confocal microscopy showing BDA- (in green) and TH-labeled fibers (in red) in the dlPAG. Arrowheads indicate double-labeled processes. Middle and lower panels: high magnification images of double-labeled processes. Arrowheads indicate double labeling. Scale bar is 10 μm.

The findings described in the previous section indicate that the A13 cell group is the main source of dopaminergic input to the dlPAG. In order to confirm the retrograde tracing data, the anterograde tracer BDA was injected into the mZI, and sections containing the mZI and the dlPAG were double stained for TH and BDA. Figure 6 shows a representative example of a BDA injection site, within the mZI, overlapping with the A13 cell group area as illustrated by the presence of BDA/TH-positive neurons. Examination of double-labeled axons and boutons within the dlPAG confirmed the presence of BDA/TH-immunoreactive profiles (Figure 7).

## Discussion

This study aimed at establishing a dopaminergic map of the dlPAG, an important brain region involved in the expression of defensive behavior and panic. The present findings provide evidence for the existence of a relatively abundant dopaminergic innervation in the dlPAG and demonstrate that this innervation originates from different areas of the diencephalon and mesencephalon. The incertohypothalamic dopamine neurons (A13) located in the rostromedial part of the zona incerta (mZI) represents the main source of dopaminergic input to the dlPAG. The extended A10 group was the second-largest source of dopaminergic input to the dlPAG; the projecting neurons were located in the PHA, the RLi and the vPAG. Projections from other hypothalamic groups (A11 and A14) were also identified.

### Methodological considerations

We determined the origin of dopaminergic afferents to the dlPAG and evaluated the relative contribution of the different dopaminergic groups. The use of retrograde tracing combined with double immunofluorescence is a reliable and sensitive method which, however, has some limitations.

The first one is related to the uptake of tracer by fibers of passage. We opted for injecting relatively large amounts of retrograde tracer by pressure injection. This allowed us to obtain large injection sites that cover an important part of the rostro-caudal extent of the dlPAG. In case of large pressure injection, however, it is possible that the tracer is taken up also by damaged passing fibers. This could lead to retrogradely labeled neurons in regions that actually do not project to the injection site (Chen and Aston-Jones, 1995). However, several arguments consolidate the results obtained from our pressure injection cases.

Firstly, we used one case (R30) in which the tracer had been delivered iontophoretically into the dlPAG. We used a relatively high current (5 μA) to obtain a tracer deposit that would give strong labeling while still being restricted to the injection site. The iontophoresis method has been shown to be more sensitive than pressure injection (Kovács and Palkovits, 2010), and this current intensity (5 μA) with CTb has been successfully utilized recently (Bienkowski and Rinaman, 2013). The comparison between this case (R30) and a case with pressure delivery of the tracer (R22) confirms the importance of the A13 and A11 dopaminergic groups in the innervation of the dlPAG.

Secondly and most importantly, we selected cases in which the patterns of CTb deposits were diverse and complementary. For instance, in cases R23 and R26, injection sites located in the more caudal part of the dlPAG are complementary to case R22. The topographies of the different cases were quite similar. Additionally, all the regions in which a high number of double-labeled neurons were found (the A13, A11, and A10 groups) have been shown to project to the dlPAG in previous studies (see below).

Therefore, if there was any uptake of CTb by passing fiber, it is likely to be rather limited. In two cases (R23 and R26), we found a low number of neurons within the A15 and A9 cell groups. Two neurons in the A15 cell group for each case, and three and four labeled neurons in the A9 cell group for R23 and R26 respectively. This labeling could be the result of CTB uptake by fibers of passage, since there are no established projections from the substantia nigra to the dlPAG (Kirouac et al., 2004).

Another drawback of tracer delivery by pressure injection is that the tracer can spread out of the limits of the region of interest. In our study, CTb deposits were also present in the SC, essentially the deep layers. The SC also contains dopaminergic fibers although less than the dlPAG (Kitahama et al., 2000) as confirmed by comparison of our TH and DBH stainings. Importantly, the analysis of case R29, in which the tracer deposit is located only in the deep layers of the SC, shows that only very few CTb/TH-positive neurons are present in dopaminergic cell groups (the A13 and A11 cell groups). CTb deposit spread also into a small portion of the dorsomedial PAG (dmPAG); therefore it is possible that some of the double-labeled neurons found are actually projecting to the dmPAG which also plays an important role in defensive behavior (Canteras, 2002).

To provide further evidence that the A13 cell group is a source of dopaminergic input to the dlPAG, the anterograde tracer BDA was injected into this area. We choose the high molecular weight BDA (10,000 mW) which is preferentially transported anterogradely (Reiner et al., 2000). Given the advantage of BDA of being able to be delivered also by pressure injection, we preferred this tracer over Phaseolus vulgaris leucoagglutinin. Thus, we could apply large injections that covered most part of the A13 group extent.

### A13 cell group

In this study, the A13 dopaminergic cell group provides most of the dopaminergic input to the dlPAG. Approximately half of the total number of CTb/TH-positive neurons was found in this dopaminergic cell group. It seems that this cell group projects along a large part of the rostro-caudal extent of the dlPAG. Indeed, among the cases selected, there were different patterns of tracer deposit along the dlPAG extent, and in all cases the A13 dopaminergic cell groups contained most of the double-labeled neurons. The A13 cell group consists of a cluster of dopaminergic neurons located in the rostromedial part of the mZI (Björklund et al., 1975) and belongs to the medial hypothalamic system (Sita et al., 2007). The connection pattern of the mZI is different from the rest of the zona incerta (ZI) which is considered to be part of the subthalamic area. Earlier studies have demonstrated the existence of important reciprocal connections between the ZI and the PAG (Shammah-Lagnado et al., 1985; Kolmac et al., 1998), and provided more detail concerning the topography of these projections and the density of the anatomical connections between the mZI/A13 and the dlPAG (Wagner et al., 1995; Geerling et al., 2010). Recently, it has been confirmed that the dmPAG and dlPAG were among the main efferents of the incerto-hypotalamic area, and particularly of the mZI containing the A13 dopaminergic cell group (Sita et al., 2007; Geerling et al., 2010). The neurochemical nature of these connections has been addressed for melanin-concentrating-hormone (MCH) and neuropeptide EI (NEI) (Elias and Bittencourt, 1997). No prior studies have specifically investigated the projection profile of dopaminergic neurons within the mZI to the PAG. Considering that dopamine is present in a distinct neuronal population than the MCH/ NEI-positive neurons (Sita et al., 2003), it is likely that it targets different neurons and participates, at least in part, in different physiological processes than the MCH/NEI-containing neurons. The A13 cell group has previously been shown to also project to the central nucleus of the amygdala (Eaton et al., 1994), which is involved in the expression of fear and other emotional behaviors (Davis, 2000; LeDoux, 2000).

To our knowledge, no study has yet attempted to relate the A13 cell group to defensive or panic-like behaviors. It is known that this dopaminergic cell group projects to other regions that belong to the defense system such as the medial preoptic area and the ventromedial hypothalamus (VMH) (Wagner et al., 1995; Dominguez and Hull, 2005; Miller and Lonstein, 2009). The VMH is a crucial center for the induction of defensive behaviors (Fuchs et al., 1985; Canteras, 2002) and dopamine agonists are known to facilitate VMH stimulation-induced defensive attack in the cat (Maeda et al., 1985; Sweidan et al., 1990). Similarly, in the medial preoptic area-anterior hypothalamus, dopamine, via the D2-receptor, facilitates affective defense behaviors in cats (Sweidan et al., 1991). It has been proposed that dopamine could facilitate defensive responses in the hypothalamus, which would in turn facilitate dlPAG action (Siegel et al., 1999). We found in the present study that the A13 dopaminergic cell group directly innervates the dlPAG. This is intriguing and suggests that dopamine could act directly on the dlPAG, thus exerting a simultaneous action on the different components of the defensive system.

### A11 cell group

In all cases studied, we found CTb/TH-positive cells in the A11 dopaminergic cell group located in the dorso-posterior hypothalamus. This cell group is in direct continuation with the A13 cell group. Our finding that the rostral and caudal CTb deposits always resulted in the presence of doubled-labeled neurons in this cell group indicates that these dopaminergic neurons project along the entire rostro-caudal extent of the dlPAG, similarly to the A13 neurons. In Beitz' study of the afferents of the different columns of the PAG (Beitz, 1982), injection of horseradish peroxidase into the dorsolateral subdivision resulted in labeled cells in the dorsal hypothalamic area and the PHA. The latter region contained retrogradely labeled neurons at both the ipsilateral and contralateral sides when the tracer had been injected into the rostral part of the dlPAG. Thus, our finding of equivalent number of CTb-labeled neuron in both hemispheres is in accordance with these observations.

### Extended A10 cell group

The extended A10 cell group is an assembly of neurons located near the ventral tegmental area, and includes the PHA, the RLi, and the vPAG (Miller and Lonstein, 2009). In the present study, we found TH/CTb-positive neurons in all three subregions.

Previous studies have shown reciprocal connections between the PHA and the PAG (Beitz, 1982; Abrahamson and Moore, 2001). The PHA mostly sends glutamatergic input to the PAG (Beart et al., 1990). However, there is a subset of PHA-neurons that contain TH (Abrahamson and Moore, 2001). These neurons most likely belong to the extended A10 dopaminergic cell group and project to the dlPAG. The PHA mediates several different functions such as nociception (Manning and Franklin, 1998) and the regulation of heart rate and blood pressure (Spencer et al., 1990) which are thought to be related to defensive reactions. Most importantly, there is evidence of its direct implication in defense and panic mechanisms. Like the posterior hypothalamus, the PHA is connected to the medial hypothalamic defense system (Canteras, 2002) and stimulation of this area provokes panic attacks in humans (Shekhar et al., 1990) and panic-like behavior in rats (Rasche et al., 2006).

The RLi is known to send very few projections to the dlPAG (Del-Fava et al., 2007). In their study, injection of the anterograde tracer Phaseolus vulgaris leucoagglutinin gave a very light labeling in the dlPAG, suggesting that a limited number of neurons in the RLi project to this column of the PAG. In the present study, we also found limited numbers of CTb/TH-positive cells in the RLi. It is therefore tempting to hypothesize that these few fibers originating from the RLi found in the Del-Fava et al. study are dopaminergic. The scarce innervation of the dlPAG suggests a very discrete and specific action by the dopaminergic neurons of the RLi. So far there is no strong evidence of an implication of the RLi in defensive behavior or panic.

Finally, we found double-labeled neurons in the vPAG. This is in agreement with another study stating that the dopaminergic neurons present in the vPAG also project within the PAG (Hache et al., 2011), and innervate the dlPAG.

## Conclusions and functional implications

Our combined anatomical tracing and transmitter identification study constitutes a first step toward a better understanding of the role of dopamine in the dlPAG and might provide a chemical and structural basis for the processing of panic-like defensive behavior. The findings demonstrate two main dopaminergic systems projecting to the dlPAG. The first is located in the hypothalamus and includes the A13, A11, and A14 cell groups. The second system is the extended A10 dopaminergic cell group of the ventral midbrain. Besides, two areas of the extended A10 dopaminergic cell group, the PHA and the vPAG, are directly involved in different aspects of defensive and panic behaviors. Interestingly, the main source of dopaminergic input to the dlPAG, the A13, is known to possibly influence the defense system of the hypothalamus.

This study demonstrates the complex organization of dopaminergic projections to the dlPAG. Further anatomical and functional studies are needed to determine whether the hypothalamic and the mesencephalic projections contact the same neurons within the dlPAG or whether they target different populations of neurons. This would help to classify the different inputs and together with behavioral and pharmacological studies provide insight concerning a potential role of dopamine in the mechanisms underlying the expression of defense and panic behavior. Several neurotransmitters have been shown to mediate the process of defensive behavior and panic in the dlPAG. Among them, the most studied are serotonin, cholecystokinin, glutamate and GABA. Recently, others neurotransmitters have been shown to participate in dlPAG functioning as well (Fogaça et al., 2012). It is likely that dopamine plays a role in regulating the expression of dlPAG-mediated panic and defensive reactions, considering the origin of its input as well as the established role of dopamine in mediating these behaviors in other parts of the defense system.

### Conflict of interest statement

The authors declare that the research was conducted in the absence of any commercial or financial relationships that could be construed as a potential conflict of interest.

## Abbreviations

3N: oculomotor nucleus
3PC: oculomotor nucleus, parvicellular part
3V: 3rd ventricle
a: artery
A11: A11 dopamine cells
A13: A13 dopamine cells
AHP: anterior hypothalamic area, posterior part
Aq: aqueduct
BDA: biotinylated dextran amine
bsc: brachium of the superior colliculus
csc: commissure of the superior colliculus
DA: dorsal hypothalamic area
Dk: nucleus of Darkschewitsch
DLPAG: dorsolateral periaqueductal gray
DMC: dorsomedial hypothalamic nucleus, compact part
DMD: dorsomedial hypothalamic nucleus, dorsal part
DMPAG: dorsomedial periaqueductal gray
DMV: dorsomedial hypothalamic nucleus, ventral part
DpG: deep gray layer of the superior colliculus
DpMe: deep mesencephalic nucleus
DpWh: deep white layer of the superior colliculus
DT: dorsal terminal nucleus of the accessory optic tract
dtgd: dorsal tegmental decussation
EW: Edinger-Westphal nucleus
f: fornix
fr: fasciculus retroflexus
IF: interfascicular nucleus
IGP: internal globus pallidus (intrapeduncular nucleus)
InC: interstitial nucleus of Cajal
InCSh: interstitial nucleus of Cajal. shell region
InG: intermediate gray layer of the superior colliculus
InWh: intermediate white layer of the superior colliculus
LPAG: lateral periaqueductal gray
Lth: lithoid nucleus
MA3: medial accessory oculomotor nucleus
MGM: medial geniculate nucleus, medial part
ml: medial lemniscus
mlf: medial longitudinal fasciculus
MPT: medial pretectal nucleus
mt: mammillothalamic tract
mtg: mammillotegmental tract
MTu: medial tuberal nucleus
mZI: medial zona incerta
opt: optic tract
PaC: paracommissural nucleus of the posterior commissure
PAG: periaqueductal gray
PaR: pararubral nucleus
PaXi: paraxiphoid nucleus of thalamus
PBP: parabrachial pigmented nucleus of the VTA
pc: posterior commissure
PCom: nucleus of the posterior commissure
PeF: perifornical nucleus
PeF: perifornical nucleus
PeFLH: perifornical part of lateral hypothalamus
PHA: posterior hypothalamic area
PHD: posterior hypothalamic area, dorsal part
PIL: posterior intralaminar thalamic nucleus
PLH: peduncular part of lateral hypothalamus
PR: prerubral field
PVG: periventricular gray
RLi: rostral linear nucleus of the raphe
RMC: red nucleus, magnocellular part
RPC: red nucleus, parvicellular part
RPF: retroparafascicular nucleus
SC: superior colliculus
SCO: subcommissural organ
scp: superior cerebellar peduncle (brachium conjunctivum)
SG: suprageniculate thalamic nucleus
SNCD: substantia nigra, compact part, dorsal tier
SNL: substantia nigra, lateral part
SNR: substantia nigra, reticular part
Stg: stigmoid hypothalamic nucleus
Su3: supraoculomotor periaqueductal gray
Su3C: supraoculomotor cap
SubB: subbrachial nucleus
SubG: subgeniculate nucleus
SubI: subincertal nucleus
Te: terete hypothalamic nucleus
ts: tectospinal tract
vPAG: ventral PAG
VTAR: ventral tegmental area, rostral part
ZI: zona incerta
ZIC: zona incerta, caudal part
ZID: zona incerta, dorsal part
ZIR: zona incerta, rostral part
ZIV: zona incerta, ventral part
Zo: zona layer of the superior colliculus.
